# Methodological Considerations Regarding the Quantification of DNA Impurities in the COVID-19 mRNA Vaccine Comirnaty^®^

**DOI:** 10.3390/mps7030041

**Published:** 2024-05-08

**Authors:** Brigitte König, Jürgen O. Kirchner

**Affiliations:** 1 Magdeburg Molecular Detections GmbH & Co. KG, 39104 Magdeburg, Germany; brigitte.koenig@medizin.uni-leipzig.de; 2 Institute of Medical Microbiology and Virology, Faculty of Medicine, University of Leipzig, 04103 Leipzig, Germany; 3 Independent Researcher, 22307 Hamburg, Germany

**Keywords:** mRNA vaccines, Comirnaty, DNA impurities, fluorescence spectroscopy, Qubit fluorometry

## Abstract

DNA impurities can impact the safety of genetically engineered pharmaceuticals; thus, a specific limit value must be set for them during marketing authorisation. This particularly applies to mRNA vaccines, as large quantities of DNA templates are used for their production. Furthermore, when quantifying the total DNA content in the final product, we must observe that, in addition to the mRNA active ingredient, DNA impurities are also encased in lipid nanoparticles and are therefore difficult to quantify. In fact, the manufacturer of the mRNA vaccine Comirnaty (BioNTech/Pfizer) only measures DNA impurities in the active substance by means of a quantitative polymerase chain reaction (qPCR), whose DNA target sequence is less than just 1% of the originally added DNA template. This means that no direct DNA quantification takes place, and compliance with the limit value for DNA contamination is only estimated from the qPCR data using mathematical extrapolation methods. However, it is also possible to dissolve the lipid nanoparticles with a detergent to directly measure DNA contamination in the final product by using fluorescence spectroscopic methods. Experimental testing of this approach confirms that reliable values can be obtained in this way.

## 1. Considerations

Among genetically engineered drugs, those with mRNA active ingredients are a special case, as their cell-free biosynthesis requires high concentrations of DNA templates, which must be removed before the products can be used as drugs. In the case of the COVID-19 mRNA vaccine Comirnaty^®^ produced by BioNTech/Pfizer (BNT162b2) (Mainz, Germany), these templates are produced by plasmids obtained from bacterial cultures [1]. Thus, Comirnaty^®^ has a special quality: DNA impurities are possible due to the manufacturing process; this may be relevant for all genetically engineered drugs, but it is otherwise rarely a problem [2]. This is due to the fact that genetically engineered active substances are predominantly proteins, which can be easily separated from DNA due to their chemical differences. Accordingly, DNA impurities in genetically engineered medicinal products have so far only been a marginal issue. However, the situation is quite different with mRNA vaccines: contaminating DNA and active ingredient mRNA are both nucleic acids and therefore chemically so similar that separation is far more difficult than separating DNA during the purification of protein active ingredients [3].

The addition of highly concentrated DNA templates, which are, in fact, linearized plasmids, to the reaction mixture that is used to produce an mRNA vaccine therefore poses a particular challenge for COVID-19 mRNA vaccines in terms of quality assurance with regard to contaminating DNA. In addition, the active substance in the form of mRNA only has low stability compared to contaminating DNA. Even exposure to room temperature can lead to the decay of RNA, whereas DNA remains stable for decades under the same conditions in the absence of degrading enzymes [4]. The lipid nanoparticles used for drug formulation, whose function is to transport the mRNA into the cells of a vaccinated person, appear to be even more sensitive. Their disintegration, which already occurs at room temperature, makes it necessary to store Comirnaty^®^ at very low temperatures. In order to achieve a shelf life that meets practical requirements, storage at −60 to −90 °C is therefore prescribed. Storage at 2 to 8 °C is also permissible but considerably shortens the product’s shelf life [5].

In order to remove the DNA templates that were added during the production process and the accompanying residues of genomic DNA of the host bacteria after the production of the mRNA active ingredient, DNA digestion with the enzyme DNase I is first carried out in the reaction mixture after the completion of cell-free mRNA synthesis. Subsequent filtration is intended to remove the resulting DNA fragments, while the mRNA is retained [6].

This may sound simple, but it must be borne in mind that the considerable chemical instability of the mRNA used can pose a problem. This is particularly because DNase digestion takes place at temperatures above 35 °C and under stirring—i.e., under conditions that could lead to significant losses of the mRNA active ingredient if the exposure lasts long enough. This means that DNA digestion must be limited in every respect, so that the mRNA yield remains economical while, at the same time, the DNA content is kept below a limit to be set in each case. This limit was set as part of the authorisation of Comirnaty^®^, with a limit value of 10 ng DNA per dose [6,7], corresponding exactly to the relevant WHO recommendations for genetically engineered medicinal products [2].

The fact that this limit value was successfully met in the production of Comirnaty^®^ was generally accepted as a given after its authorisation. However, this dogma had to be reconsidered after the US scientist Kevin McKernan and his team made it public that they had found large quantities of DNA impurities in Comirnaty^®^ [8], most of which were present in quantities that were several hundred times higher than the applicable limit of 10 ng DNA per dose. Other scientists followed with their own results, including the Canadian group led by David Speicher [9] and the US cancer researcher Phillip Buckhaults, who presented his findings to the South Carolina Senate [10].

Is it therefore possible that the DNA quantifications carried out for Comirnaty^®^ as part of batch testing were incorrect? In order to verify this, it is first necessary to examine the methodical procedure employed. This question primarily stems from a European Medicines Agency (EMA) document that was created as part of the approval procedure and dates from 19 November 2020 [6]. This source states that DNA quantification takes place in the active substance after DNase digestion and filtration have been carried out. This document also states that the method of choice for this DNA analysis is a quantitative polymerase chain reaction, abbreviated as qPCR, wherein the target sequence is only 69 base pairs of the total 7824-base-pair-long DNA template, whereby the sequence of the T7 promoter is integrated, an important step for the transcription process for the production of the mRNA active substance. Therefore, only the presence of this sequence is checked; the remaining 7755 base pairs, and thus 99% of the template, and any remaining genomic DNA of the host bacterium remain undetermined.

According to further official information from the German government [11], a theoretical DNA content is extrapolated from the measured value, obtained via this qPCR measurement, and compared with the limit value of 10 ng DNA per dose. What this means in detail is explained in the EMA document from 19 November 2020, which has already been cited above [6]. According to this document, a dilution series is produced with the linearized plasmid, which serves as a DNA template for in vitro transcription and which, in turn, is to be measured using qPCR. A standard curve is generated from the data obtained when measuring the dilution series. Finally, the measurement results of the active substance samples are mathematically compared with this standard curve through extrapolation. However, it is not clear from the description of the above-mentioned EMA document that the processes to which the DNA templates, i.e., the linearized plasmids, are subjected during the manufacturing process are taken into account in any way. This applies in particular to in vitro transcription, in addition to DNase and proteinase digestion and filtration, processes that remove small DNA and protein fragments into which the DNA templates and the added enzymes have been degraded. Anything that affects the linearized plasmids during the manufacturing process does not appear to be taken into account when creating the standard curve. However, this would be necessary to allow the standard curve to actually reflect what the qPCR measures. This applies in particular to what actually happens during DNase digestion. With this in mind, the following questions are of the utmost importance:

1. Which DNA fragments are specifically formed during DNase digestion, and is the qPCR target sequence actually degraded proportionally to the entirety of the remaining fragments of the linearized plasmids? Are distinct sequences of the linearized plasmids degraded more frequently or significantly less frequently by DNase than others?
2. What influence do the in vitro transcription conditions have on the sequence of the T7 promoter, which is part of the qPCR target sequence? It should be considered that the T7 promoter has a special affinity for the polymerase used, so the target sequence may be at least partially masked by the polymerase or its fragments resulting from proteinase digestion and therefore be potentially unmeasurable using qPCR.
3. Does the target sequence, which is only 69 base pairs long, actually remain in a quantity that is proportional to the other sequences remaining after DNase digestion and the subsequent filtration steps? If the proportionality is not given, any extrapolation is bound to be wrong.

These questions show that when using DNA quantification via qPCR, it is difficult to obtain reproducible values that correspond to the actual ratios for the given question, i.e., whether the limit value of 10 ng DNA per dose of the end product is adhered to.

Against this background, it is no surprise that the European Pharmacopoeia 2.6.35. Quantification And Characterization of Residual Host-Cell DNA [12] states that qPCR is the method of choice for the quantification of specific DNA sequences, while the measurement of total DNA is not assigned to qPCR but to other methods.

A further communication from the German Federal Government [7] also states that batch testing in Europe is carried out according to a protocol [13] published by the European Directorate for Quality in Medicine, EDQM. This document confirms the following: apart from the singular measurement at the active substance level conducted by the manufacturer, no further experimental DNA quantification is carried out for the vaccine, especially not for the final product, not even in the context of official batch testing.

This approach raises the question of how this can be justified. The answer can also be found in an official statement made by the German government [11]. According to this statement, the quantification of DNA impurities should be carried out in the active substance, as a measurement in the ready-to-use vaccine could be disturbed by the lipid nanoparticles that it contains, which could lead to incorrect values. At first glance, this sounds acceptable. However, further examination of the EDQM protocol shows that the mRNA active ingredient—a nucleic acid like DNA—is quantified despite the lipid nanoparticles contained in the final product. But if the quantification of mRNA is not disturbed by lipid nanoparticles, this should, in principle, also apply to the quantification of DNA due to the common properties of nucleic acids. Hence, how is it that the quantification of RNA in the end product is accepted as feasible by state institutions, while the quantification of DNA at the same level of production, i.e., in the ready-to-use vaccine, is not?

Documents published by the Australian Therapeutic Goods Administration (Australian Government, Department of Health) provide important facts for answering this question. Firstly, there is a batch release document for Comirnaty^®^ that has been issued by Sciensano, the National Laboratory of Belgium [14]. This document reveals that RNA is determined in the final vaccine using a fluorescence spectrometric method. Another document published by the Australian Therapeutic Goods Administration [15] reveals what this method is, as it provides validation data concerning this method. According to this document, the RNA-specific fluorescent dye RiboGreen^®^ is used for mRNA quantification in Comirnaty^®^ at the level of the finished product. This dye binds highly specifically to RNA, resulting in fluorescence that is proportionally dependent on the amount of RNA that is present and can be measured. RiboGreen^®^, in turn, is one of the fluorescent dyes that are part of the fluorometric Quant-iT^®^ system produced by ThermoFisher Scientific (Dreieich, Germany) [16].

Since Quant-iT^®^ measurements are carried out using the analysers that are regularly available in quality control laboratories, it is necessary to validate a method specifically on the device used. Such a validation was recorded in the aforementioned documentation published by the Australian Government [15]. These validation data also reveal that for RNA quantification, it is necessary to disintegrate the lipid nanoparticles in order to release the mRNA that is bound in them and make it accessible for measurement, wherein this disintegration of the lipid nanoparticles is in turn carried out using the detergent Triton-X-100 (final concentration 1%).

In addition to the RNA-specific RiboGreen^®^, the analogue but DNA-specific fluorescent dye PicoGreen^®^ is also available as an alternative, so that DNA quantification in the final vaccine can be carried out just as reliably as RNA quantification after the disintegration of the lipid nanoparticles using Triton-X-100 [15]. In addition to the Quant-iT^®^ system, ThermoFisher Scientific also offers the Qubit^®^ system for specific quantification using fluorescent dyes. While Quant-iT^®^ enables a higher sample throughput with standard laboratory equipment (microtitre plate readers), Qubit^®^ is the method of choice in laboratories where no equipment for extensive routine tests is available and a comparatively low sample throughput is expected [17]. Qubit^®^ uses an automated fluorescence spectrometer that can be combined with standardised Qubit^®^ test kits. These Qubit^®^ kits are optimised for either RNA or DNA quantification, and standardised kits for protein quantification are also available [18]. Due to this versatility, Qubit^®^ is standard equipment in many molecular biology laboratories. The excellent selectivity of Qubit^®^ has been extensively validated and documented by the manufacturer, as has the low influence of impurities contained in the samples. In particular, it was proven that high quantities of RNA do not alter Qubit DNA quantification, while the Nanodrop^®^ spectrophotometer failed in this regard [19]. Qubit^®^ therefore has an advantage over Quant-iT^®^: certain test validations that are required when using Quant-iT^®^ on the standard device used can be omitted due to the manufacturer’s calibration [18]. The two systems, Quant-iT^®^ and Qubit^®^, therefore correspond to each other in terms of functionality. This means that both Quant-iT^®^ and Qubit^®^ can distinguish DNA and RNA with the highest reliability using highly specific binding fluorescent dyes.

It was therefore necessary to investigate the practical suitability of Qubit for the quantification of total DNA in Comirnaty. To this end, a series of experiments were carried out, involving both RNA and DNA quantification (details are given in the Supplementary Materials, including data on possible confounding factors).

**Figure 1:**
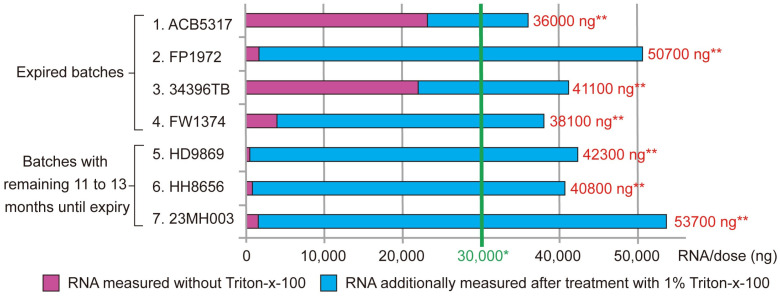
Quantification of total RNA in batches of Comirnaty^®^ using Qubit^®^ fluorometry without and with the addition of Triton-X-100 as a detergent to disintegrate the lipid nanoparticles contained in the vaccine formulation. The measured values shown as bars in the figure refer to the total RNA content in ng per dose of ready-to-use diluted Comirnaty^®^. In all batches, it was found that the measured RNA value increased considerably after treatment with Triton-X-100. As expected, this could only be a consequence of the dissolution of the lipid nanoparticles and the resulting release of the RNA that was bound in them. In batches 1 to 4, which had all expired, it was found that after treatment with Triton-X-100, between 36 and 97% of the maximum measured RNA value had become accessible for measurement due to the dissolution of the lipid nanoparticles, while in batches 5 to 7, which still had a shelf life of 11 to 13 months, this value was between 97 and 99% of the total RNA. Two of four expired batches may have largely disintegrated even without treatment with a detergent, whereas this was only caused by Triton-X-100 in the batches with a longer shelf life. Irrespective of this, however, very high RNA values were measurable in all batches after Triton-X-100 treatment, significantly exceeding the target value for one dose of 30 µg (30,000 ng). * Target value for one dose: 30,000 ng (30 µg) of RNA (300 µL of ready-to-use Comirnaty). ** Total RNA ng/dose after treatment with 1% Triton-X-100.

Figure 1 shows the results of measuring mRNA with Qubit^®^ in seven batches of Comirnaty^®^ without and after treatment with Triton-X-100. Four batches were already expired, while three batches had a remaining shelf life of 11 to 13 months. The results clearly show that the treatment of Comirnaty^®^ with Triton-X-100 leads to a significant increase in RNA values. In the specific series of tests carried out, this effect appears to depend partly on whether the batch had already expired at the time of measurement or whether it still had a long shelf life. This means that in two of four expired batches, also without Triton-X-100, over 50% of the total RNA was measurable. This suggests that in expired batches, the lipid nanoparticles are disintegrated, even without Triton-X-100, whereas in vaccines with a long shelf life, 97 to 99% of the total RNA was only measurable after the lipid nanoparticles were dissolved with Triton-X-100 (further details are included in the Supplementary Materials).

However, if the mRNA active substance was quantifiable using fluorescence spectrometry in the final mRNA vaccine after it was treated with Triton-X-100, this should also be possible for the DNA impurities as an integrated part of the batch testing of Comirnaty^®^.

**Figure 2:**
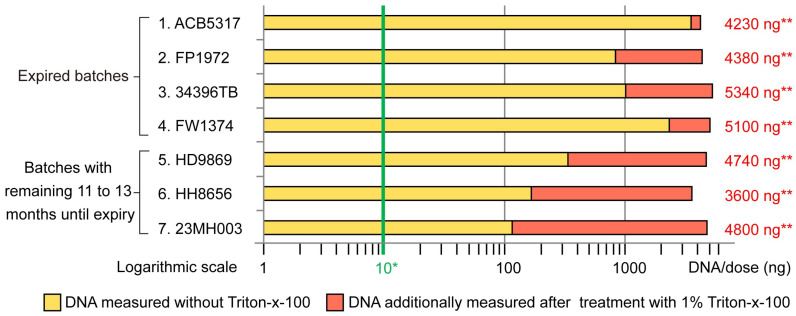
Quantification of total DNA in batches of Comirnaty^®^ using Qubit^®^ fluorometry without and with the addition of Triton-X-100 as a detergent to disintegrate the lipid nanoparticles contained in the vaccine formulation. The measured values shown as bars in the figure refer to the total DNA content in ng per dose of ready-to-use diluted Comirnaty^®^. These measurement results must be compared with the limit value for the total DNA content of 10 ng DNA per dose for Comirnaty^®^. One dose consists of 300 µL of ready-to-use vaccine. In all batches, it was found that the measured DNA value increased considerably after treatment with Triton-X-100. As expected, this could only be a consequence of the dissolution of the lipid nanoparticles and the resulting release of the DNA that was bound in them. In batches 1 to 4, which had all expired, it was found that after treatment with Triton-X-100, between 16 and 81% of the maximum measured DNA value had become accessible for measurement due to the dissolution of the lipid nanoparticles, while in batches 5 to 7, which still had a shelf life of 11 to 13 months, this was even as high as between 93 and 97% of the total DNA. This indicates that the lipid nanoparticles from expired batches may have largely disintegrated even without treatment with a detergent, whereas this was only caused by Triton-X-100 in the batches with a longer shelf life. Irrespective of this, however, very high DNA values were measurable in all batches after Triton-X-100 treatment, with these values ranging from 360 to 534 times the permissible DNA limit or 3600 to 5340 ng DNA per dose. * Threshold of 10 ng of DNA/dose (300 µL of ready-to-use Comirnaty). ** Total DNA ng/dose after treatment with 1% Triton-X-100.

In order to verify this assumption, corresponding DNA quantifications in ready-to-use diluted Comirnaty^®^ batches with and without Triton-X-100 were conducted. Figure 2 shows the results (further details are included in the Supplementary Materials): if Comirnaty^®^ is treated with Triton-X-100, the result is a significant increase in DNA values for some of the batches but not for others. In the specific series of tests carried out, this effect appears to depend on whether the batch had already expired at the time of measurement or whether there was still a long shelf life of 11 or more months at the time of measurement. This suggests, as already found via mRNA testing, that in expired batches, the lipid nanoparticles are at least partly disintegrated even without Triton-X-100, whereas in vaccines with a long shelf life, they are still largely intact and include the DNA impurities, so they are not fully accessible for measurement due to this compartmentalisation.

## 2. Conclusions

The available information and data indicate that the ready-to-use mRNA vaccine Comirnaty contains DNA impurities that exceed the permitted limit value by several hundred times and, in some cases, even more than 500 times, and that this went unnoticed because the DNA quantification carried out as part of batch testing only at the active substance level appears to be methodologically inadequate when using qPCR, as explained above. Because of the conditions during the production of the mRNA active substance of Comirnaty, the applied qPCR is designed so that a massive under-detection of DNA impurities appears to be the result. Here, we have to remember that qPCR is matchless if specific DNA sequences are being quantified, but this is not the case if the aim is the quantification of the total DNA content. However, DNA contamination in Comirnaty is about total DNA, regardless of the sequences that it contains. Accordingly, it can be assumed that a fluorescence spectrometric measurement of the total DNA in the end product, analogous to the quantification of the mRNA active ingredient, a process that is, in fact, carried out in the end product, is not associated with a risk of under-detecting DNA contaminations but rather provides reliable values and thus satisfies the required level of drug safety.

Against this background, experimental testing of the total DNA contained in the ready-to-use diluted vaccine Comirnaty^®^ via fluorescence spectrometric measurement, which is to be carried out by the authorities as part of the legal mandate for official batch testing, appears to be essential. Why this was systematically omitted by the European control laboratories according to the statements by the German Federal Government cited above should therefore be the subject of extensive expert discussions and reconsiderations.

Further, it should also be taken into account that DNA impurities in Comirnaty^®^ are apparently integrated into the lipid nanoparticles and are thus transported directly into the cells of a vaccinated person, just like the mRNA active ingredient. What this means for the safety risks, particularly the possible integration of this DNA into the human genome, i.e., the risk of insertional mutagenesis, should be a secondary focus of the discussion required, which must go far beyond what could have been considered years before the so unexpected introduction of mRNA pharmaceuticals into the global market.

## Data Availability

The original contributions presented in the study are included in the article/Supplementary Material, further inquiries can be directed to the corresponding author.

## Supplementary Materials

The following supporting information can be downloaded at https://www.mdpi.com/article/10.3390/mps7030041/s1: Table S1. Qubit^®^ fluorometry: Quantification of total RNA in batches of Comirnaty^®^ without and with Triton-X-100. Table S2. Qubit^®^ fluorometry: Quantification of total DNA in batches of Comirnaty^®^ without and with Triton-X-100. Table S3. Qubit^®^ fluorometry: Influence of DNA concentration on DNA quantification. Table S4. Qubit^®^ fluorometry: Influence of Triton-X-100 on DNA quantification. Table S5. Qubit^®^ fluorometry: DNA quantification in the mRNA vaccine in the presence of 1% Triton-X-100.
