# Expression of Concern: König, B.; Kirchner, J.O. Methodological Considerations Regarding the Quantification of DNA Impurities in the COVID-19 mRNA Vaccine Comirnaty^®^. *Methods Protoc.* 2024, *7*, 41

**DOI:** 10.3390/mps8040068

**Published:** 2025-06-25

**Authors:** Methods and Protocols Editorial Office

**Affiliations:** MDPI, Grosspeteranlage 5, 4052 Basel, Switzerland; mps@mdpi.com; Tel.: +41-61-683-77-34

Following publication [1], concerns were raised to the Editorial Office regarding the appropriateness of methodology and validity of the data presented in this published protocol.

The *Methods and Protocols* Editorial Board evaluated the concerns and the response from the authors and decided to initiate a Comment and Reply process as per MDPI policy on Updating Published Papers (https://www.mdpi.com/ethics#_bookmark30 accessed on 13 June 2025) and as per the Committee on Publication Ethics guidance on Handling Post Publication Critiques (https://publicationethics.org/guidance/flowchart/handling-post-publication-critiques accessed on 13 June 2025).

Before the conclusion of this process, the Editorial Office and Editorial Board became aware of a peer-reviewed publication detailing the critiques [2]. As per our Comment and Reply policies (https://www.mdpi.com/ethics#_bookmark29 accessed on 13 June 2025), this Comment could no longer be accepted as Comments should not reiterate previously published disagreements (Updating Published Papers policy). As a result, the Editorial Office and Editorial Board have decided to publish this Expression of Concern for the purpose of informing the readers of the debate and acknowledging the concerns related to this published protocol. The Editorial Office will provide an update on this situation and announce any post-publication modification deemed necessary.
