# Supplementary material for: Methodological Considerations Regarding the Quantification of DNA Impurities in the COVID-19 mRNA Vaccine Comirnaty®
Source: Methods Protoc. 2024 May 8;7(3):41. doi: 10.3390/mps7030041 (PMC11130870; doi:10.3390/mps7030041)
Supplement: Supplementary file 1 [file mps-07-00041-s001.zip › mps-2928338-supplementary.pdf]

methods and protocols

MDPI

Supplementary Materials

Methodological Considerations Regarding the Quantification of DNA Impurities in the COVID-19 mRNA Vaccine Comirnaty®

Brigitte König [1,2] and Jürgen O. Kirchner [3]

Experiment 1—Qubit® fluorometry: Influence of Triton-X-100 on RNA measurement in Comirnaty®

The detergent Triton-X-100 is suitable for dissolving lipid nanoparticles. This makes it possible to test how the dissolution of lipid nanoparticles affects the DNA content measured in the vaccine[1]. Therefore, the influence of such a treatment with Triton-X-100 was tested regarding its influence on the quantification of DNA in Comirnaty® using Qubit® fluorometry.

For testing purposes, the conducting Laboratory Magdeburg Molecular Detections GmbH & Co. KG received various samples of different batches of the mRNA vaccine Comirnaty® (BNT162b2) from BioNTech/Pfizer. All these samples were provided as original sealed vials by official vaccination centres that stated that the samples had been deep-frozen or stored at 2°C-8°C until dispatch to Magdeburg Molecular Detections GmbH & Co. KG. The samples were dispatched in compliance with the cold chain (2 to 8 °C), which was established on arrival. The samples were stored at 2 to 8°C until they were analysed.

The samples were diluted in a ready-for-use state according to the applicable provisions before analysis with Qubit® according to the official instructions for use provided by the manufacturer. According to method documentation published by the Australian Therapeutic Goods Administration, the samples to be tested after the dissolution of lipid nanoparticles* were spiked with Triton-X-100 so that the final concentration was 1 %. Then, the samples were incubated for 15 minutes at roomtemperature.

RNA quantification was performed by employing fluorometry using the Qubit® RNA HS assay kit (ThermoFisher Scientific, Germany, catalogue number Q32852; batch 2397757) and a Qubit® 3 fluorometer were used according to the manufacturer's instructions.

Result

The treatment of a vaccine with Triton-X-100 led to a significant increase in RNA values. This effect appeared to depend partly on whether the batch had already expired at the time of measurement (batches 1 and 3) or whether it still had a long shelf life of 11 or more months at the time of measurement (batches 5 to 7). This suggests that in expired batches, the lipid nanoparticles were disintegrated even without Triton-X-100, whereas in vaccines with a long shelf life, they are still largely intact, so the RNA is not accessible for measurement due to this compartmentalisation.

Table S1. Qubit® fluorometry: Quantification of total RNA in batches of Comirnaty® (ready-to-use dilution) without and with Triton-x-100 (final concentration 1%). One representative experiment out of three is shown.

[1] Australian Government, Department of Health, Therapeutic Goods Administration, 2021: Quantitation of Total and Percent Encapsulated RNA in PF-07302048 Lipid Nanoparticles by RiboGreen Fluorescence Worksheet, dated 10 Aug 2021 and Pfizer - BNT162b2 - Quantification of Total and Percent Encapsulated RNA in PF-07302048 Lipid Nanoparticles by RiboGreen Fluorescence Assay Data, dated 01 Nov 2021, both together published by the Australian Therapeutic Goods Administration, https://www.tga.gov.au/sites/default/files/foi-3390-07.pdf.

No	Batch Designation	Expiry Date	Without Triton-X-100				With 1% Triton-X-100	
			RNA ng/µL	RNA ng/dose (300 µL)	Remaining Time Until the Expiry Date Will be Reached	Months Exceeding the Expiry Date	RNA ng/µL	RNA ng/dose (300 µL)
1	ACB5317	02/2022	76.8	23040	–	18 months	120	36000
2	FP1972	04/2022	5.12	1536	–	16 months	169	50700
3	34396TB	06/2022	73.6	22080	–	14 months	137	41100
4	FW1374	09/2022	13.1	3930	–	11 months	127	38100
5	HD9869	10/2024	1.5	450	11 months	–	141	42300
6	HH8656	12/2024	4.28	728	12 months	–	136	40800
7	23MH003	01/2025	5.36	1608	13 months	–	179	53700

Experiment 2—Qubit® fluorometry: Influence of Triton-X-100 on DNA measurement in Comirnaty®.

DNA quantification was performed by means of fluorometry using the Qubit 1X dsDNA HS assay kit (ThermoFisher Scientific, Germany, catalogue number Q33230; batch 2339927) and a Qubit 3 fluorometer according to the manufacturer’s instructions. The measurement results must be compared with the limit value for the total DNA content of 10 ng of DNA per dose, as applicable to Comirnaty®. One dose consists of 300 µL of ready-to-use vaccine (for further details, please see Experiment 1).

Result

Dissolving the lipid nanoparticles with Triton-X-100 resulted in a significantly increased DNA value, so it can be assumed that RNA was released from the lipid nanoparticles.

Table S2. Qubit® fluorometry: Quantification of total DNA in batches of Comirnaty® (ready-to-use dilution) without and with Triton-x-100 (final concentration 1%). One representative experiment out of three is shown.

No	Batch Designation	Expiry Date	Without Triton-X-100				With 1% Triton-X-100		Additional DNA released through treatment with Triton-X-100 in % of the total DNA found
			DNA ng/µL	DNA ng/dose	Remaining time until the expiry date will be reached	Months exceeding the expiry date	DNA ng/µL	DNA ng/dose (300 µL)	
1	ACB5317	02/2022	11.8	3540	–	18 months	14.1	4230	16
2	FP1972	04/2022	2.78	834	–	16 months	14.6	4380	81
3	34396TB	06/2022	3.38	1014	–	14 months	17.8	5340	81
4	FW1374	09/2022	7.78	2334	–	11 months	17.0	5100	54
5	HD9869	10/2024	1.12	336	11 months	–	15.8	4740	93
6	HH8656	12/2024	0.556	166.8	12 months	–	12.0	3600	95
7	23MH003	01/2025	0.389	115.8	13 months	–	16.0	4800	98

Experiment 3—Qubit® fluorometry: Influence of DNA concentration on DNA measurement in Comirnaty®

To determine the influence of DNA concentration on DNA measurement, different amounts of DNA were added to vaccine samples (ready-to-use dilution).

Result

No relevant influence of the DNA concentration on the accuracy of the DNA measurement could be determined.

Table S3. Qubit® fluorometry: Influence of DNA concentration on DNA quantification. One representative experiment out of three is shown.

Added DNA Standard		DNA Measured in the Sample (ng/µL)			
		Water		Comirnaty® Batch HD9869	
Dilution	Increasing the DNA Content by Adding the Standard in the Following Amounts	Measured Value	Measured Value Minus Added DNA Standard	Measured Value	Measured Value Minus Added DNA Standard
0 control	0	0 (“too low”)	0	1.04	1.04
undiluted	10 ng/µL	9.72	0	10.7	0.7
1:2	5 ng/µL	4.28	0	4.68	0
1:5	2 ng/µL	1.67	0	2.08	0.08
1:10	1 ng/µL	0.972	0	1.39	0.39
1:100	0.1 ng/µL	0.104	0	0.504	0.404

Experiment 4—Qubit® fluorometry: Influence of Triton-X-100 on DNA measurement in Comirnaty®

The detergent Triton-X-100 is suitable for dissolving lipid nanoparticles. It can therefore be used to test how the dissolution of lipid nanoparticles affects the DNA content measured in the vaccine using fluorometry. To determine whether Triton-X-100 affects these measurements, the total DNA was first quantified with Qubit® fluorometry in aqueous DNA standards of different concentrations with and without 1% Triton X-100 (for further details, see experiment 1). In a parallel test, the same standard concentrations were added to the vaccine, and the total DNA was also quantified without and with 1% Triton X-100.

Result

Triton-X-100 does not influence DNA measurements. If a vaccine is treated with Triton-X-100, this leads to a significant increase in DNA values. This indicates that the otherwise intact lipid nanoparticles remove the DNA impurities from the measurement. If the lipid nanoparticles are disintegrated with Triton-X-100, these DNA impurities are also released and accessible for Qubit® measurement.

Sub-experiment 4a—Qubit® fluorometry: Influence of Triton-X-100 on DNA quantification control.

Sub-result 4a: The accuracy of the DNA measurement in the DNA standard (in the absence of the vaccine) is not affected by Triton-X-100

Table S4. Qubit® fluorometry: Influence of Triton-x-100 on DNA quantification. One representative experiment out of three is shown.

DNA Standard		DNA Measured in the Sample (ng/µL)			
		Without Triton-X-100		With 1% Triton-X-100	
Dilution	DNA Content	Measured Value	Measured Value Minus Added DNA Standard	Measured Value	Measured Value Minus Added DNA Standard
Control	0	0 (“too low”)	0	0.0232	0
undiluted	10 ng/µL	9.48	0	9.56	0
1:2	5 ng/µL	6.00	0,1	6.64	1.64
1:5	2 ng/µL	2.03	0	1.92	0
1:10	1 ng/µL	0.98	0	0.804	0
1:100	0.1 ng/µL	0.112	0	0.124	0

Sub-experiment 4b—Qubit® fluorometry: Influence of adding 1% Triton-X-100 into Comirnaty® (ready-to-use dilution) on DNA quantification

Sub-result 4b: Dissolving the lipid nanoparticles with Triton-X-100 resulted in a significantly increased DNA value, so it can be assumed that DNA was released from the lipid nanoparticles.

Table S5. Qubit® fluorometry: DNA quantification in the mRNA vaccine (ready-to-use dilution) in the presence of 1% Triton-x-100. One representative experiment out of three is shown.

DNA Standard		DNA Measured in the Vaccine Comirnaty® Batch HH8656 (ng/µL)			
Dilution	Increasing the DNA Content by Adding the Standard in the Following Amounts	Without Triton X-100		With 1% Triton X-100	
		Measured Value	Measured Value Minus Added DNA Standard	Measured Value	Measured Value Minus Added DNA Standard
Control	0	0.556	0,556	12.0	12.0
undiluted	10 ng/µL	8.32	0	21.2	11.2
1:2	5 ng/µL	4.88	0	15.6	10.6
1:5	2 ng/µL	1.62	0	13.0	11.0
1:10	1 ng/µL	0.884	0	11.6	10.6
1:100	0.1 ng/µL	0.331	0.231	10.0	9.9
